# Preventive Dentistry

**Published:** 1902-12-15

**Authors:** C. M. Wright

**Affiliations:** Cincinnati


THE
### DENTAL REGISTER.

Vol. LVI. December 15, 1902. No. 12.

### PREVENTIVE DENTISTRY.

BY C. M. WRIGHT, D.D.S., CINCINNATI.

There can be no question as to the fact that dentistry is founded on hygiene, and that from the beginning dentists have been as deeply interested, theoretically, in preventing as in curing disease.

From the beginning it has been held that "clean" teeth are far less liable to disease than are neglected ones, and yet from some faults in habits of practice, rather than in theory, the dental profession has slighted a fundamental operation in prevention and devoted itself to more attractive and ingenius methods of cure and *partial* prevention.

Dr. D. D. Smith, of Philadelphia, has lately called the attention of the profession of America to this neglect, and pointed out some of the important results of a thorough and *frequent* polishing by hand, with orange wood and pumice, of all the exposed surfaces of the teeth. He has also forcefully advocated a method of practice which is strictly logical in its relation to *real* prevention.

Personally, I entirely agree with Dr. Smith, but, recognizing the difficulty of changing habits of practice and of educating the people to accept the change, I suggested a few months ago a scheme for the forming of a sub-specialty in dentistry. I urged the colleges to establish a special partial course for the training of cultured women in this one line, viz., the polishing of the teeth and the care of the mouth. The paper was printed in the April, 1902 number of the *International Dental Journal*, of Philadelphia, and kindly commented upon by Dr. Truman in an able editorial in the same journal. I have received a number of letters from dentists on the subject, and from such men as Drs. Samuel A. Hopkins, of Boston; G. A. Mills, of New York; Levi C. Taylor, of Hartford, generally favoring the plan.

There seem to be legal and other obstacles in the way, and fears of the innovation that might be made by partially educated class of sub-specialists. I tried to answer a few of the objections in a paper read before the Kentucky State Dental Society, in May, 1902. If we cannot control the situation by some such plan as that which I have suggested, we must perform these operations ourselves if we are true to our convictions. The operations require considerable time and skill, and heretofore, from our habits of slurring and our wrong methods of adjusting a compensating fee, we have not been able to afford the proper service.

Can we modify our practice? We are visited semi-annually or oftener by numbers of our careful patients for "examination." Our examinations mean too often only a search for carious cavities, which we expect at a later appointed time to treat and fill. If we find two or six cavities we reserve the proper time, and charge for each filling as an operation, or for the one, two or three hours of the sitting. We may even spend a part of these hours in removing accretions of tartar and in rapidly polishing the surfaces exposed to sight with brush-wheels, discs and points of our engines. Then if we "itemize our services," even in our own minds, we say: "One gold filling in bicuspid, so much; one "t.g." in crown of molar, so much; removing tartar, so much." The last item is the obnoxious stumbling-block. It is cleansing a *set of teeth*," not polishing, or treating by massage a central incisor, or a cuspid, or molar. It is not as Dr. Smith insists on calling it, a "prophylactic operation" upon each tooth liable to disease. This prophylactic operation upon one or several individual teeth would be the scientific method of applying at frequent intervals an irritant that is not simply for the removal of adhesive plaques and accretions of fermentable substances, but a stimulation of physiological forces within the tooth and about the root or roots of the tooth, in the pulp and pericemental membrane, as well as the epithelia of the gingival border. These forces offer natural resistance to external injurious agencies. When these forces are dormant we have, without doubt, the pre-disposing causes of more than one disease of the tooth and its surrounding tissues.

Caries of the dentin and "Rigg's disease," are dependent upon two etiologic factors—the predisposing and the specific exciting cause, and in this prophylactic treatment by polishing and massage, both causes are recognized and the surgeon has *prevention* in sight as his object. This is reasonable.

Suppose as a profession we come to a full appreciation of the value to the human family of this one fundamental proposition, viz., that if a tooth be skillfully rubbed or polished on every exposed surface once every fortnight, adhesions will be kept away, the effects of fermentations will be obliterated, pulps and nourishing membranes will be stimulated to healthy and normal function, and caries and septic gingivitis will be positively prevented. Would it be worth while to impress the doctrine on our patients and change the methods and habits of our daily practice to meet the requirements? Many of us would lay aside our gold foils and porcelains and much of the paraphernalia of our office, and make a specialty of this *prophylactic surgery*. Preventive dentistry, so-called, would prevent diseases —dental and oral—but would increase enormously the demands on the dentist's time and services. This is why I advocated the establishment of a class of sub-specialists.

The field of operating, in schools and homes and in our offices, is world-wide, and the laborers have not yet come forth.

I have offered two suggestions and am thoroughly imbued with the importance of them. I regret the inability to forcibly express myself and make the pictures live in your imaginations as they do in mine.

The first of these is to establish a special course in dental colleges for the teaching of prophylactic dental surgery as a specialty. The second is to individualize the teeth and to learn to treat each tooth singly, and to consider the operation on each tooth as worthy of a fee.

I shall not enter into the question of cost to the patient at this time, though I think each of us can prove by a study of past records of patients whom we have treated from their childhood that if we had pursued the plan suggested the expense to them would have been no greater, and the results infinitely more satisfactory.

